# Are We Always Prepared for Urgent Coronary Interventions in Complex Adult Congenital Heart Disease Patients?

**DOI:** 10.1155/cric/5534523

**Published:** 2026-04-30

**Authors:** Shailendra Upadhyay, Whitney Fairchild, Olga T. Salazar

**Affiliations:** ^1^ Division of Pediatric Cardiology, Holtz Children′s Hospital, University of Miami Miller School of Medicine, Miami, FL, USA, miami.edu; ^2^ Division of Pediatric Cardiology, Connecticut Children′s, Hartford, CT, USA; ^3^ Division of Pediatric Cardiology, Vanderbilt Children′s Hospital, Nashville, TN, USA

**Keywords:** adult congenital heart disease, coronary dissection, Taussig–Bing anomaly

## Abstract

The adult congenital heart disease population is on the rise, and coronary artery angiography may be required for evaluation of epicardial coronary arteries in these patients. A patient with complex congenital heart disease, including the Taussig‐Bing anomaly, repaired with the Rastelli operation, developed iatrogenic right coronary artery dissection during selective angiography. This was successfully managed in a challenging procedure involving extensive stenting of the right coronary artery with a good outcome.

## 1. Introduction

The adult congenital heart disease (ACHD) population has been progressively growing with a continued increase in its prevalence [1]. ACHD patients may have coronary disease by virtue of congenital anomalies, external compression, aorta/aortic root intervention, coronary artery reimplantation, or acquired atherosclerosis [2, 3]. Diagnostic or interventional coronary angiography is sometimes required to address myocardial ischemia concerns in these patients. Unanticipated, rare complications may occur during selective coronary angiography. Here, we present this case to highlight the need for emergency preparedness for coronary angiography when planned in ACHD patients. This case report has been prepared in accordance with the institutional ethics guidelines. The case report involved a review of deidentified data. It was exempt from the requirements of patient consent per the institutional review board guidelines.

## 2. Case

**Figure 1:**
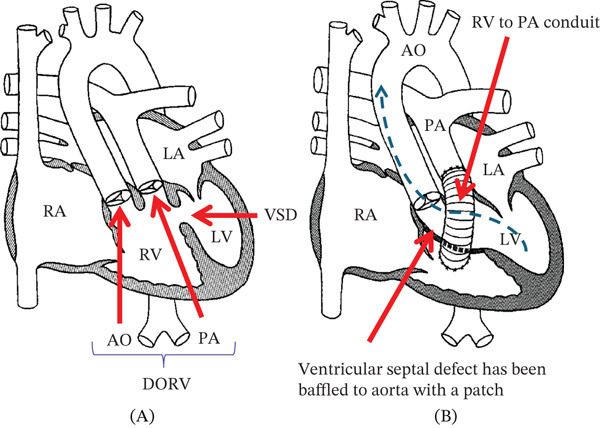
(A) Cartoon of unrepaired anatomy of Taussig–Bing Anomaly (double outlet right ventricle [DORV] with transposition of the great arteries and ventricular septal defect [VSD]). (B) Repaired anatomy with VSD baffled to the aorta (AO) and placement of a right ventricle (RV) to the pulmonary artery (PA) conduit. Abbreviations: RA, right atrium; LA, left atrium; RV, right ventricle; LV, left ventricle.

**Figure 2:**
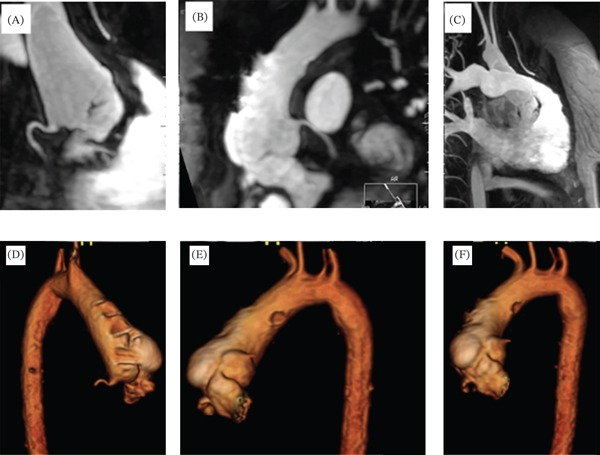
Cardiac MRI demonstrating anatomy of the aorta. (A) Origin of the right coronary artery. (B) Origin of the left coronary artery. (C) Relationship of the right ventricular outflow tract with the aorta. (D–F) Various three‐dimensional reconstructed views of the aorta/aortic root. Note the bulbous irregularities due to previous patch augmentation of the aorta.

Cardiac catheterization was performed in a 37‐year‐old woman with double outlet right ventricle, ventricular septal defect, and D‐transposition of the great arteries (Taussig–Bing anomaly). Her initial palliation included atrial septectomy and pulmonary artery banding as a neonate. At approximately 5 years of age, she underwent repair with a Rastelli operation involving enlargement of her ventricular septal defect, baffle closure of the ventricular septal defect to the aorta, and placement of a right ventricle to pulmonary artery conduit. She subsequently underwent two replacements of the right ventricle to pulmonary artery conduit at 15 and 29 years of age for conduit stenosis and insufficiency (Figure 1). At her most recent conduit revision, an aorta‐to‐right atrial fistula originating near the aortic root/sinus region was also closed. Coronary artery origins were not compromised during the fistula repair. Symptoms noted at her recent follow‐up included progressive exertional shortness of breath with NYHA Class II symptoms (decline in peak VO_2_ on exercise test at 17 mL/kg/m), functional decline, and fatigue. Her baseline ECG demonstrated a sinus rhythm at 68 BPM with first‐degree AV block (PR interval of 206 ms), right bundle branch block (QRS duration 155 ms), and a left axis deviation. The ECG had not significantly changed in the past 12 years. The right bundle branch block, however, made interpretation of ST‐T segments unreliable. Her transthoracic echocardiogram demonstrated no significant right ventricle to pulmonary artery conduit insufficiency or stenosis (peak instantaneous gradient of 14 mmHg). The shortening fraction was noted to have declined from 27.5% to 24.1% in 1 year. Cardiac MRI demonstrated a left ventricular ejection fraction of 49%, not significantly different from her cardiac MRI in 2008 (Figure 2). No concerns for right ventricle to the pulmonary artery conduit obstruction were noted. The aortic root was noted to be bulbous. The left coronary system was noted to course under the right pulmonary artery (Figure 2). Up to 145 ms of right ventricle–left ventricle dysynchrony was noted on cardiac MRI.

We decided to proceed with a diagnostic cardiac catheterization to look for any hemodynamic causes of her exertional dyspnea. Selective coronary angiography was also planned to rule out any external coronary compression from the right ventricle to the pulmonary artery conduit.

Cardiac catheterization was performed by an interventional cardiologist with additional training in ACHD and structural heart disease interventions at an adult institution.

Hemodynamic data demonstrated a cardiac index of 2.7 L/min/m^2^, a pulmonary capillary wedge pressure of 8 mmHg, a mean pulmonary artery pressure of 16 mmHg, and a right ventricle to the pulmonary artery conduit gradient of 4 mmHg with normal right ventricle and right atrial pressures. Pulmonary vascular resistance was within the normal range at 1.7 indexed Woods units. No left ventricular outflow obstruction was present. Selective coronary angiography was performed after the hemodynamic measurements, which demonstrated a somewhat unusual course of a nonobstructed, nondominant left coronary system.

The origin of the right coronary artery (RCA) was unusual due to the previous aortic root surgery, which was performed to repair the aorta to right atrium fistula. The origin was patulous and redundant (shepherd′s crook origin). The RCA was a dominant system (Figure 2A). Upon engagement of the RCA, the catheter sat deeply into the roof of the shepherd′s crook origin of the vessel. This resulted in iatrogenic RCA dissection. The complete spiral dissection extended distally with complete RCA occlusion. The patient developed an acute inferolateral ST‐segment elevation myocardial infarction, chest discomfort, and heart block. Emergency transvenous pacing, inotropic support, and intra‐aortic balloon counter pulsations were initiated. Attention was then diverted to an intervention on the dissected RCA, performed by a coronary interventionist. Extreme difficulty was encountered in attempts to cannulate the RCA, given the rotated and bulbous ascending aorta and aortic root. Initial attempts at entering the true lumen distally from the false lumen were unsuccessful. Attempts at entering the vessel more proximally near its dissection were unsuccessful. With a fine cross catheter, a pilot 200 wire was manipulated into the right ventricular marginal branch near the mid portion of the RCA (confirmed with contrast injection). The wire was then gradually withdrawn and then advanced distally into the right posterolateral branch with subsequent advancement of the fine cross catheter at this location. The pilot wire was exchanged for a 300‐cm pro coronary guidewire. The proximal coronary dissection was identified with an intravascular ultrasound. The vessel lumen measured at 5–5.5 mm at this location, where a 5 × 3.8‐mm ultrabare metal stent was deployed. This resulted in residual TIMI Grade 1 flow in the RCA. Two other 5 × 3.8‐mm bare metal stents were placed proximally to the cover to the point of the RCA ostium. The vessel flow remained poor, and subsequently, the entire RCA was stented.

**Figure 3:**
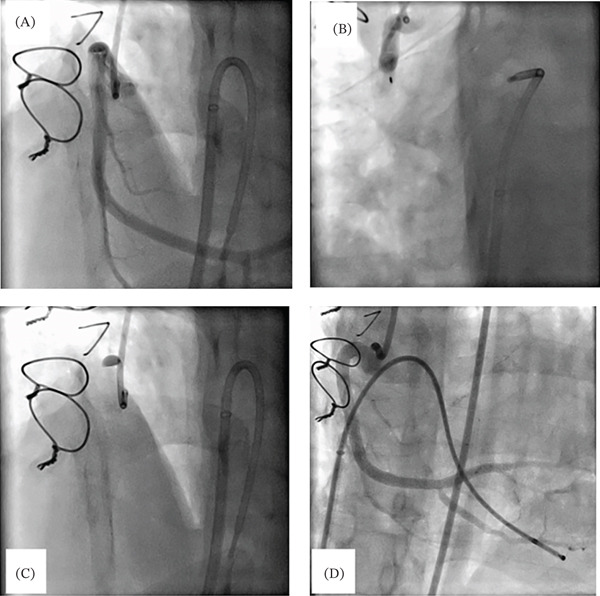
Selective right coronary artery (RCA) angiography. (A) Selective RCA angiography with no coronary stenosis. (B, C) Contrast hang‐up due to iatrogenic RCA dissection. (D) Selective right coronary angiography after extensive RCA stenting with good patency. Note the temporary pacing catheter in the right ventricle that was inserted due to the development of heart block from RCA dissection.

A total of seven stents (four bare metal and three drug‐eluting) were placed across the length of the RCA. The procedure also involved reopening of the posterior right descending artery. Aspiration thrombectomy and intracoronary vasodilator infusion were performed as well. The result was excellent with no coronary stenosis and TIMI Grade 3 flow Figure 3.

The patient was later managed in the coronary care unit. The ST elevations resolved, and the intra‐aortic balloon pump was discontinued. Her ventricular function remained unchanged on the day after the coronary intervention. She did well and was discharged home the next day. She remains stable after approximately 3 years of follow‐up with her primary adult congenital cardiologist, with return of her ventricular function to baseline. Follow‐up CTA revealed patent right and left coronary systems.

## 3. Discussion

The Taussig–Bing anomaly was first described in 1949, and it represents the spectrum of double outlet right ventricle with ventricular septal defect and D‐transposition of the great arteries [4, 5]. The anatomic defect involves rotation of aortic cusps, and coronary artery origins may become difficult to engage with conventional coronary catheters (Figure 2). Rastelli operation for the Taussig–Bing anomaly involves baffling the ventricular septal defect to the aorta and placement of a right ventricle to the pulmonary artery conduit.

Coronary artery anatomy, course, and origins may be abnormal to various degrees in ACHD patients [2, 3, 6]. Some unique situations also include external coronary compression from conduits [7]. This has been demonstrated in tetralogy of Fallot patients with right ventricle to pulmonary artery conduit undergoing transcatheter pulmonary valve replacement [8].

ACHD patients become subject to acquired coronary artery disease with their advancing age. The prevalence of coronary artery disease in the ACHD population has been reported to be similar to that of the general population, and atherosclerotic coronary artery disease is known to occur [9, 10]. There have also been reports of acute coronary syndrome in a 24‐year‐old male with a single ventricle anomaly [11]. ACHD patients have a much higher prevalence of metabolic syndromes, and added cardiovascular risks may add to the development of premature coronary artery disease [12–14]. Clinical concerns, such as unexplained dyspnea and possible coronary compression in repaired ACHD patients with conduits, may necessitate evaluation of the coronaries. Cardiac CT angiography may noninvasively provide anatomic details of the coronary anatomy [15]. However, dynamic external coronary obstruction and hemodynamic data needed in certain ACHD patients may require cardiac catheterization. In the case presented here, we performed a diagnostic cardiac catheterization to obtain hemodynamic data and rule out any coronary obstruction, particularly with symptoms suggestive of compensated heart failure (NYHA Class II dyspnea and fatigue) and left ventricular systolic dysfunction. Although cardiac MRI provided important information regarding the distorted aortic root and coronary origins, CT coronary angiography may also have been a useful noninvasive modality in this patient to further define coronary anatomy, exclude distal coronary disease, and better anticipate challenges related to selective coronary cannulation.

Selective coronary angiography is very commonly performed for the evaluation of epicardial coronary artery disease in adults. A rare, yet dreaded, complication of coronary angiography is coronary artery dissection, with an incidence below 0.1% [16, 17]. Coronary artery dissections are managed with coronary stent implantation. There are no reported cases of coronary dissection in complex ACHD patients. The anomalous origin of the right coronary system (shepherd′s crook origin) likely contributed to the dissection on a background of previous surgical intervention on the aorta to treat an aorta‐to‐right atrium fistula.

Successful coronary interventions in patients with congenital heart disease in both pediatric and ACHD populations have been performed in close collaboration with pediatric and adult interventional cardiologists [18, 19]. Several ACHD centers are based at children′s hospitals, and congenital heart catheterizations are preferred/recommended to be performed by an ACHD interventional cardiologist. However, such centers may not be best equipped to deal with acute coronary emergencies if the hospitals are not attached to or part of an adult hospital. If a selective diagnostic coronary catheterization is planned, it is essential to discuss the optimal location for cardiac catheterization. It is also quite important to understand the anatomy and various prior repairs that may have been performed on the coronaries or the ascending aorta. This will enable the coronary interventionist to best prepare for unanticipated complications. The combined team effort with the adult congenital cardiologist, ACHD interventional cardiologist, and coronary interventionalist leads to a prompt and successful management of extensive RCA dissection in this patient.

## 4. Conclusion

Coronary dissection in ACHD patients is rare but can pose significant challenges in management due to difficulty with manipulating routine catheters for coronary intervention, given an anatomically abnormal aortic root and CA origins. A multidisciplinary team approach, including an interventional ACHD specialist and a coronary interventionalist, is warranted. The ideal location for catheterization in a pediatric versus an adult catheterization laboratory is likely to be program‐specific, based on local resources and expertise; however, close collaboration among team members seems prudent. A multicenter study looking at coronary intervention outcomes in this population may highlight further data to deal with coronary complications in ACHD patients.

## Funding

No funding was received for this manuscript

## Conflicts of Interest

None of the authors have a conflict of interest to disclose

## Data Availability Statement

Data sharing not applicable to this article as no datasets were generated or analysed during the current study.
